# Myonuclear dynamics with age and exercise: shaping up for a good time

**DOI:** 10.1113/JP284303

**Published:** 2023-02-10

**Authors:** Cory M. Dungan

**Affiliations:** ^1^ Department of Health, Human Performance and Recreation Baylor University Waco TX USA

**Keywords:** ageing, exercise, myonuclei, nuclear shape

For many years, the structure and function of myonuclei in response to ageing and exercise has been of great interest to the exercise community (Bagley et al., 2025). From their permanency in response to detraining (Murach et al., 2020) to their postmitotic nature in mature skeletal muscle (Borowik et al., 2023), myonuclei have been examined closely to understand how they adapt to ageing and exercise. Although the field lacks a consensus on various aspects of myonuclear dynamics, there is a growing consensus that myonuclei can change their shape and position in response to exercise training; however, it is unclear whether this process is affected by ageing. In this issue of the *Journal of Physiology*, Battey et al. (2025) provide more clarity to this question by examining myonuclear shape and structure in skeletal muscle from young and old, sedentary and exercised humans.

Changes in myonuclear shape with age were originally characterized by Haithcock et al. (2005), who described alterations in myonuclear architecture in muscle from old *Caenorhabditis elegans*. Furthermore, they demonstrated the importance of myonuclear lamins in maintaining myonuclear shape and in lifespan (Haithcock et al., 2005). Since then, studies from numerous laboratories, including the Peterson laboratory (Murach et al., 2020), have reported changes in the structure of myonuclei using various cell and animal model systems of age and exercise. The study by Battey et al. (2025) provides new insight into change in myonuclear dynamics, because they provide an in‐depth characterization of myonuclei from young and old humans. The authors demonstrate that exercise training, regardless of age, increased the sphericity of myonuclei, in addition to reducing the aspect ratio. In old humans, exercise also increased myonuclear lamin A deposition, which, based on work from Haithcock et al. (2005), could help to facilitate long‐term muscle and whole‐body health. To provide further clarity on their findings in humans, the authors then performed an exercise‐training study in mice and showed greater abundance of lamin A after 8 weeks of exercise training, confirming their findings in humans. Battey et al. (2025) also showed higher expression of SUN2, a nuclear membrane protein that is crucial for nuclear structure and motility, along with greater sphericity and rigidity in myonuclei from exercise‐trained mice. Although the work by Battey et al. (2025) did not identify a significant difference with age, work from the Larsson laboratory suggests that there could be sex‐ and fibre type‐specific differences in myonuclear dynamics that need to be accounted for (Cristea et al., 2010). Taken together, exercise training alters myonuclear shape and stiffness regardless of age, which could have long‐term implications for delaying sarcopenia or augmenting muscle function in older individuals.

Perhaps the real question is, where do we go from here? It is clear that exercise is invaluable for the preservation of lifespan and healthspan, delaying muscle atrophy and improving physical function, along with treating various metabolic diseases; however, the impact of myonuclear dynamics on ageing is a bit less obvious. In the paper by Battey et al. (2025), the authors describe how increased myonuclear stiffness can negatively affect chromatin organization and transcription factor translocation, potentially leading to altered gene expression. Moreover, they discuss how elongation of myonuclei could lead to chromatin stretching and increased the expression of genes associated with muscle atrophy, in addition to increasing the susceptibility of the nuclear envelope to rupture. In a broader context, the idea of promoting myonuclear sphericity is intriguing for various other aspects of skeletal muscle ageing, such as sarcopenia, anabolic resistance and blunted muscle regeneration. This could also be applicable to other disease states and conditions that are associated with dysfunction in skeletal muscle, including cancer/chemotherapy‐induced cachexia, obesity and metabolic disease, and muscle stem cell dysfunction. Lastly, work in rodents indicates that these changes in myonuclear shape are not permanent, because myonuclei will return to a more protracted shape after a period of detraining (Murach et al., 2020), which could have implications in athletic performance and beyond. Although the work by Battey et al. (2025) is an exciting next step for those interested in myonuclei, there are many avenues to take and much work to be done.

It is becoming increasingly clear that the integrity of the myonucleus and nuclear envelope is important for long‐term health, and exercise seems to be the best medicine for maintaining myonuclear dynamics. Whether you are a superstar athlete or a weekend warrior trying to stave off the ‘dad body’, one thing is certain; if you want to have a good time, you'd better keep your myonuclear dynamics in line.

## Additional information

### Competing interests

None.

### Author contributions

Sole author.

### Funding

None.

## Supporting information


Peer Review History
