# Supplementary material for: Myonuclear dynamics with age and exercise: shaping up for a good time
Source: J Physiol. 2023 Feb 10;603(13):3859–60. doi: 10.1113/JP284303 (PMC12306386; doi:10.1113/JP284303)
Supplement: Supplementary file 1 — Peer Review History [file TJP-603-3859-s001.pdf]

The Journal of Physiology JP

Myonuclear dynamics with age and exercise: Shaping up for a good time

Cory M Dungan
DOI: 10.1113/JP284303

Corresponding author(s): Cory Dungan (cory_dungan@baylor.edu)

The following individual(s) involved in review of this submission have agreed to reveal their identity: Matthew J Stroud (Referee #1)

Review Timeline:

Submission Date:	13-Jan-2023
Editorial Decision:	19-Jan-2023
Revision Received:	19-Jan-2023
Editorial Decision:	23-Jan-2023
Revision Received:	23-Jan-2023
Editorial Decision:	25-Jan-2023
Revision Received:	25-Jan-2023
Accepted:	30-Jan-2023

Senior Editor: Michael Hogan

Reviewing Editor: Kevin Murach

Transaction Report:

(Note: With the exception of the correction of typographical or spelling errors that could be a source of ambiguity, letters and reports are not edited. Depending on transfer agreements, referee reports obtained elsewhere may or may not be included in this compilation. Referee reports are anonymous unless the Referee chooses to sign their reports.)

Dear Dr Dungan,

Re: JP-P-2023-284303 "Myonuclear dynamics with age and exercise: Shaping up for a good time" by Cory M Dungan

Thank you for submitting your manuscript to The Journal of Physiology. It has been assessed by a Reviewing Editor and by 1 expert referee and we are pleased to tell you that it is acceptable for publication following minor revision.

Please advise your co-authors of this decision as soon as possible.

The referee reports are copied at the end of this email.

Please address all the points raised and incorporate all requested revisions or explain in your Response to Referees why a change has not been made. We hope you will find the comments helpful and that you will be able to return your revised manuscript within 2 weeks. If you require longer than this, please contact journal staff: jp@physoc.org.

Your revised manuscript should be submitted online using the link in your Author Tasks: Link Not Available. This link is accessible via your account as Corresponding Author; it is not available to your co-authors. If this presents a problem, please contact journal staff (jp@physoc.org). Image files from the previous version are retained on the system. Please ensure you replace or remove any files that are being revised.

If you do not wish to submit a revised version of your manuscript, you must inform our journal staff (jp@physoc.org) or reply to this email to request withdrawal. Please note that a manuscript must be formally withdrawn from the peer review process at one journal before it may be submitted to another journal.

TRANSPARENT PEER REVIEW POLICY: To improve the transparency of its peer review process, The Journal of Physiology publishes online (as supporting information) the peer review history of all articles accepted for publication. Readers will have access to decision letters, including Editors' comments and referee reports, for each version of the manuscript, as well as any author responses to peer review comments. Referees can decide whether or not they wish to be named on the peer review history document.

LANGUAGE EDITING AND SUPPORT FOR PUBLICATION: If you would like help with English language editing, or other article preparation support, Wiley Editing Services offers expert help, including English Language Editing, as well as translation, manuscript formatting, and figure formatting at www.wileyauthors.com/eeo/preparation. You can also find resources for Preparing Your Article for general guidance about writing and preparing your manuscript at www.wileyauthors.com/eeo/prepresources.

REVISION CHECKLIST:
Upload a full Response to Referees file. To create your 'Response to Referees' copy all the reports, including any comments from the Senior and Reviewing Editors, into a Microsoft Word, or similar, file and respond to each point, using font or background colour to distinguish comments and responses and upload as the required file type.

Please upload two versions of your manuscript text: one with all relevant changes highlighted and one clean version with no changes tracked. The manuscript file should include all tables and figure legends, but each figure/graph should be uploaded as separate, high-resolution files.

You may also upload:
- 'Potential Cover Art' for consideration as the issue's cover image.
- Appropriate Supporting Information (Video, audio or data set: see https://jp.msubmit.net/cgi-bin/main.plex?form_type=display_requirements#supp).

We look forward to receiving your revised submission.

If you have any queries, please reply to this email and we will be pleased to advise.

Yours sincerely,

Michael C. Hogan
Senior Editor
The Journal of Physiology
https://jp.msubmit.net
http://jp.physoc.org
The Physiological Society
Hodgkin Huxley House
30 Farringdon Lane
London, EC1R 3AW
UK

http://www.physoc.org
http://journals.physoc.org

EDITOR COMMENTS

Reviewing Editor:

Thank you for agreeing to write this commentary. Your work has been evaluated by the authors of the original work and has been deemed accurate and interesting. I agree with this assessment. The authors have made some suggested changes that we encourage you to implement.

I also have a few comments to add. First, please remove reference to Josh Allen (an American football player who is likely unknown to most people outside of America) from the text. Also, it may be worth incorporating insights from Cristea et al, who studied myonuclear shape with aging: https://onlinelibrary.wiley.com/doi/full/10.1111/j.1474-9726.2010.00594.x

----

REFEREE COMMENTS

Referee #1:

Many thanks for your excellent review of our manuscript and its context.

Please see attached file for specific comments.

Title: Myonuclear dynamics with age and exercise: Shaping up for a good time

Authors: Cory M. Dungan[1,*]

Affiliations: [1]Department of Health, Human Performance and Recreation; Baylor University

* Indicates corresponding author

Cory M. Dungan, PhD
Baylor University
Robbins College of Health and Human Sciences
Department of Health, Human Performance and Recreation
Cory_Dungan@baylor.edu
Office: (254) 710-4042

Key Words: Myonuclei, Exercise, Aging, Nuclear Shape

For many years, the structure and function of myonuclei in response to aging and exercise has been of great interest to the exercise community. From their permanency in response to detraining (Murach et al., 2020) to their post-mitotic nature in mature skeletal muscle (Borowik et al., 2023), myonuclei have been closely examined to better understand how they adapt to aging and exercise. Although the field lacks a consensus on various aspects of myonuclear dynamics, there is a growing consensus that myonuclei can change their shape and position in response to exercise training; however, it is unclear if this process is affected by aging. In this issue of the *Journal of Physiology*, Battey et al. (Battey et al., 2023) provide more clarity to this question by examining myonuclear shape and structure in skeletal muscle from young and old, sedentary and exercised, humans.

Changes in myonuclear shape with age ~~was~~were originally characterized by Haithcock et al., ~~where they describe~~who described alterations in myonuclear architecture in muscle from old *C. elegans* (Haithcock et al., 2005). Furthermore, they ~~demonstrate~~demonstrated the importance of myonuclear ~~laminins~~Lamins in ~~not only~~ maintaining myonuclear shape ~~but also in~~as well as lifespan (Haithcock et al., 2005). Since then, work from numerous labs, including our own (Murach et al., 2020), have reported changes in the structure of myonuclei using various cell and animal model systems of age and exercise. The work by Battey at al. provides new insight into change in myonuclear dynamics, as they provide one of, if not the~~,~~ first, in-depth characterization of myonuclei from young and old humans. ~~Globally, the~~The authors demonstrate that exercise training, regardless of age, increased the sphericity~~ of myonuclei~~, in addition to reducing the aspect ratio~~.~~ of myonuclei. In old humans, exercise also increased myonuclear ~~Laminin~~Lamin A deposition, which, based on work from Haithcock et al., could help facilitate long-term muscle and whole-body health (Haithcock et al., 2005). To provide further clarity ~~to~~on their findings in humans, the authors then performed an exercise training study in mice and showed greater abundance of Lamin A following 8 weeks of exercise training, confirming their findings in humans. Battey et al. also show higher expression of SUN2, a nuclear membrane protein that crucial for nuclear structure and motility (Jahed et al., 2018), along with greater sphericity and rigidity in myonuclei from exercise-trained mice (Battey et al., 2023). Taken together, exercise training ~~preserves~~alters myonuclear shape and stiffness regardless of age, which could have long-term implications for delaying sarcopenia or augmenting muscle ~~hypertrophy~~function in older individuals.

Formatted: Highlight

Perhaps the real question is, where do we go from here? It’s clear that exercise is invaluable for the preservation of lifespan and healthspan, delaying muscle atrophy and improving physical function, along with treating various metabolic diseases; however, the impact of myonuclear dynamics on aging is a bit less obvious. In Battey et al., the authors describe how increased myonuclear stiffness can negatively affect chromatin organization and transcription factor translocation, potentially leading to altered gene expression. Moreover, they discuss how ~~elongated~~elongation of myonuclei ~~leads~~could lead to chromatin stretching and ~~can increase~~increased the expression of genes associated with muscle atrophy, in addition to increasing the susceptibility of the nuclear envelope to ~~rupturing~~rupture. In a broader context, the idea of promoting myonuclear sphericity is intriguing for various other aspects of skeletal muscle aging such as sarcopenia,

anabolic resistance, and blunted muscle regeneration. This could also be applicable to other disease states and conditions that are associated with dysfunction in skeletal muscle, including cancer/chemotherapy-induced cachexia, obesity and metabolic disease, and muscle stem cell dysfunction. Lastly, our work in rodents indicate that these changes in myonuclear shape are not permanent, as myonuclei will return to a more protracted shape following a period of detraining (Murach et al., 2020), which could have implications in athletic performance and beyond. Although the work by Battey et al. is an exciting next step for those interested in myonuclei, there are many avenues to take and much work to be done.

It's becoming increasingly clear that the integrity of the myonucleus and nuclear envelope is important for long-term health, and exercise seems to be the best medicine for maintaining myonuclear dynamics. So whether you're leap-frogging defenders like Josh Allen or a weekend warrior trying to stave off the dad body, one thing is for certain; if you want to have a good time, you better keep your myonuclear dynamics in line.

References:

Battey, E., Ross, J. A., Hoang, A., Wilson, D. G. S., Han, Y., Levy, Y., . . . Stroud, M. J. (2023). Myonuclear alterations associated with exercise are independent of age in humans. *J Physiol*. doi:10.1113/JP284128

Borowik, A. K., Davidyan, A., Peelor, F. F., 3rd, Voloviceva, E., Doidge, S. M., Bubak, M. P., . . . Miller, B. F. (2023). Skeletal Muscle Nuclei in Mice are not Post-mitotic. *Function (Oxf), 4*(1), zqac059. doi:10.1093/function/zqac059

Haithcock, E., Dayani, Y., Neufeld, E., Zahand, A. J., Feinstein, N., Mattout, A., . . . Liu, J. (2005). Age-related changes of nuclear architecture in Caenorhabditis elegans. *Proc Natl Acad Sci U S A, 102*(46), 16690-16695. doi:10.1073/pnas.0506955102

Jahed, Z., Vu, U. T., Fadavi, D., Ke, H., Rathish, A., Kim, S. C. J., . . . Mofrad, M. R. K. (2018). A molecular model for LINC complex regulation: activation of SUN2 for KASH binding. *Mol Biol Cell, 29*(16), 2012-2023. doi:10.1091/mbc.E18-04-0266

Murach, K. A., Mobley, C. B., Zdunek, C. J., Frick, K. K., Jones, S. R., McCarthy, J. J., . . . Dungan, C. M. (2020). Muscle memory: myonuclear accretion, maintenance, morphology, and miRNA levels with training and detraining in adult mice. *J Cachexia Sarcopenia Muscle, 11*(6), 1705-1722. doi:10.1002/jcsm.12617

Funding

N/A

Acknowledgements

I would like to thank my friend and colleague, Dr. Kevin Murach, for the many conversations on myonuclear biology during our time as postdocs at the University of Kentucky Center for Muscle Biology.

Author Contributions

C.M.D. prepared and reviewed the manuscript.

Conflicts of Interest

All authors declare no conflicts of interests.

I would like to thank the reviewer(s) for the positive review of this Perspectives article. I have incorporated all of the suggested changes to the text, including the removal of the NFL superstar quarterback, Joshua P. Allen of the Buffalo Bills. Further, I have added text referencing the manuscript by Cristea et al. and their work on myonuclear domain in humans.

Dear Dr Dungan,

Re: JP-P-2023-284303R1 "Myonuclear dynamics with age and exercise: Shaping up for a good time" by Cory M Dungan

Thank you for submitting your manuscript to The Journal of Physiology. It has been assessed by a Reviewing Editor and we are pleased to tell you that it is acceptable for publication following minor revision.

The review report is copied at the end of this email.

Please address all the points raised and incorporate all requested revisions or explain in your Response to Referees why a change has not been made. We hope you will find the comments helpful and that you will be able to return your revised manuscript within 2 weeks. If you require longer than this, please contact journal staff: jp@physoc.org.

Your revised manuscript should be submitted online using the link in your Author Tasks: Link Not Available. This link is accessible via your account as Corresponding Author; it is not available to your co-authors. If this presents a problem, please contact journal staff (jp@physoc.org). Image files from the previous version are retained on the system. Please ensure you replace or remove any files that are being revised.

If you do not wish to submit a revised version of your manuscript, you must inform our journal staff (jp@physoc.org) or reply to this email to request withdrawal. Please note that a manuscript must be formally withdrawn from the peer review process at one journal before it may be submitted to another journal.

TRANSPARENT PEER REVIEW POLICY: To improve the transparency of its peer review process, The Journal of Physiology publishes online (as supporting information) the peer review history of all articles accepted for publication. Readers will have access to decision letters, including Editors' comments and referee reports, for each version of the manuscript, as well as any author responses to peer review comments. Referees can decide whether or not they wish to be named on the peer review history document.

LANGUAGE EDITING AND SUPPORT FOR PUBLICATION: If you would like help with English language editing, or other article preparation support, Wiley Editing Services offers expert help, including English Language Editing, as well as translation, manuscript formatting, and figure formatting at www.wileyauthors.com/eeo/preparation. You can also find resources for Preparing Your Article for general guidance about writing and preparing your manuscript at www.wileyauthors.com/eeo/prepresources.

REVISION CHECKLIST:
Upload a full Response to Referees file. To create your 'Response to Referees' copy all the reports, including any comments from the Senior and Reviewing Editors, into a Microsoft Word, or similar, file and respond to each point, using font or background colour to distinguish comments and responses and upload as the required file type.

Please upload two versions of your manuscript text: one with all relevant changes highlighted and one clean version with no changes tracked. The manuscript file should include all tables and figure legends, but each figure/graph should be uploaded as separate, high-resolution files.

You may also upload:
- 'Potential Cover Art' for consideration as the issue's cover image.
- Appropriate Supporting Information (Video, audio or data set: see https://jp.msubmit.net/cgi-bin/main.plex?form_type=display_requirements#supp).

We look forward to receiving your revised submission.

If you have any queries, please reply to this email and we will be pleased to advise.

Yours sincerely,

Michael C. Hogan
Senior Editor
The Journal of Physiology
https://jp.msubmit.net
http://jp.physoc.org
The Physiological Society
Hodgkin Huxley House
30 Farringdon Lane
London, EC1R 3AW
UK
http://www.physoc.org
http://journals.physoc.org

EDITOR COMMENTS

Reviewing Editor:

The Cristea paper is not cited in the text or references. I'm guessing this was an error given there is mention of the Larsson laboratory. I don't think it is customary for J Phys style to list all the authors of a paper in the text (see the Bagley et al. reference). Also, the author states that Battey is the first in-depth characterization of myonuclei with aging, but Cristea et al. did look at aging in their work.

Just these minor tweaks and we can make a final decision. Thank you.

1st Confidential Review **19-Jan-2023**

I would like to thank the reviewer(s) for the positive review of this Perspectives article. I have added the reference for Cristea et al. and fixed the multi-name citation for Bagley et al. Also, I have removed the mention of Battey et al. being the first characterization of myonuclei in aging muscle.

Dear Dr Dungan,

Re: JP-P-2023-284303R2 "Myonuclear dynamics with age and exercise: Shaping up for a good time" by Cory M Dungan

Thank you for submitting your manuscript to The Journal of Physiology. It has been assessed by a Reviewing Editor and we are pleased to tell you that it is acceptable for publication following minor revision.

The review report is copied at the end of this email.

Please address all the points raised and incorporate all requested revisions or explain in your Response to Referees why a change has not been made. We hope you will find the comments helpful and that you will be able to return your revised manuscript within 2 weeks. If you require longer than this, please contact journal staff: jp@physoc.org.

Your revised manuscript should be submitted online using the link in your Author Tasks: Link Not Available. This link is accessible via your account as Corresponding Author; it is not available to your co-authors. If this presents a problem, please contact journal staff (jp@physoc.org). Image files from the previous version are retained on the system. Please ensure you replace or remove any files that are being revised.

If you do not wish to submit a revised version of your manuscript, you must inform our journal staff (jp@physoc.org) or reply to this email to request withdrawal. Please note that a manuscript must be formally withdrawn from the peer review process at one journal before it may be submitted to another journal.

TRANSPARENT PEER REVIEW POLICY: To improve the transparency of its peer review process, The Journal of Physiology publishes online (as supporting information) the peer review history of all articles accepted for publication. Readers will have access to decision letters, including Editors' comments and referee reports, for each version of the manuscript, as well as any author responses to peer review comments. Referees can decide whether or not they wish to be named on the peer review history document.

LANGUAGE EDITING AND SUPPORT FOR PUBLICATION: If you would like help with English language editing, or other article preparation support, Wiley Editing Services offers expert help, including English Language Editing, as well as translation, manuscript formatting, and figure formatting at www.wileyauthors.com/eeo/preparation. You can also find resources for Preparing Your Article for general guidance about writing and preparing your manuscript at www.wileyauthors.com/eeo/prepresources.

REVISION CHECKLIST:
Upload a full Response to Referees file. To create your 'Response to Referees' copy all the reports, including any comments from the Senior and Reviewing Editors, into a Microsoft Word, or similar, file and respond to each point, using font or background colour to distinguish comments and responses and upload as the required file type.

Please upload two versions of your manuscript text: one with all relevant changes highlighted and one clean version with no changes tracked. The manuscript file should include all tables and figure legends, but each figure/graph should be uploaded as separate, high-resolution files.

You may also upload:
- 'Potential Cover Art' for consideration as the issue's cover image.
- Appropriate Supporting Information (Video, audio or data set: see https://jp.msubmit.net/cgi-bin/main.plex?form_type=display_requirements#supp).

We look forward to receiving your revised submission.

If you have any queries, please reply to this email and we will be pleased to advise.

Yours sincerely,

Michael C. Hogan
Senior Editor
The Journal of Physiology
https://jp.msubmit.net
http://jp.physoc.org
The Physiological Society
Hodgkin Huxley House
30 Farringdon Lane
London, EC1R 3AW
UK
http://www.physoc.org
http://journals.physoc.org

EDITOR COMMENTS

Reviewing Editor:

Congratulations on the nice commentary. Considering the related work we performed (Murach et al. 2020 JCSM) was performed in the Peterson laboratory, I request that you change the language regarding this. With this change, we can make a rapid decision on acceptability. Thank you.

2nd Confidential Review **23-Jan-2023**

Thank you for correcting this error. I have changed it so the appropriate lab has been credited for the work.

3rd Revision - Editorial Decision 30-Jan-2023

Dear Dr Dungan,

Re: JP-P-2023-284303R3 "Myonuclear dynamics with age and exercise: Shaping up for a good time" by Cory M Dungan

Thank you for agreeing to write this article and for reviewing the original manuscript. This is a nice contribution to the literature. Congratulations!

We are pleased to tell you that your Perspective has been accepted for publication in The Journal of Physiology.

TRANSPARENT PEER REVIEW POLICY: To improve the transparency of its peer review process, The Journal of Physiology publishes online (as supporting information) the peer review history of all articles accepted for publication. Readers will have access to decision letters, including Editors' comments and referee reports, for each version of the manuscript, as well as any author responses to peer review comments. Referees can decide whether or not they wish to be named on the peer review history document.

The last Word (or similar) version of the manuscript provided will be used by the Production Editor to prepare your proof. When this is ready you will receive an email containing a link to Wiley's Online Proofing System. The proof should be thoroughly checked and corrected as promptly as possible.

Authors should note that it is too late at this point to offer corrections prior to proofing. The accepted version will be published online, ahead of the copy edited and typeset version being made available. Major corrections at proof stage, such as changes to figures, will be referred to the Editors for approval before they can be incorporated. Only minor changes, such as to style and consistency, should be made at proof stage. Changes that need to be made after proof stage will usually require a formal correction notice.

All queries at proof stage should be sent to: TJP@wiley.com.

Are you on Twitter? Once your paper is online, why not share your achievement with your followers? Please tag The Journal (@jphysiol) in any tweets and we will share your accepted paper with our 30,000 followers!

Yours sincerely,

Michael C. Hogan
Senior Editor
The Journal of Physiology
https://jp.msubmit.net
http://jp.physoc.org
The Physiological Society
Hodgkin Huxley House
30 Farringdon Lane
London, EC1R 3AW
UK
http://www.physoc.org
http://journals.physoc.org

P.S. - You can help your research get the attention it deserves! Check out Wiley's free Promotion Guide for best-practice recommendations for promoting your work at www.wileyauthors.com/eeo/guide. You can learn more about Wiley Editing Services which offers professional video, design, and writing services to create shareable video abstracts, infographics, conference posters, lay summaries, and research news stories for your research at www.wileyauthors.com/eeo/promotion.

IMPORTANT NOTICE ABOUT OPEN ACCESS: To assist authors whose funding agencies mandate public access to published research findings sooner than 12 months after publication, The Journal of Physiology allows authors to pay an Open Access (OA) fee to have their papers made freely available immediately on publication.

The Corresponding Author will receive an email from Wiley with details on how to register or log-in to Wiley Authors Services where you will be able to place an order.

You can check if your funder or institution has a Wiley Open Access Account here: https://authorservices.wiley.com/author-resources/Journal-Authors/licensing-and-open-access/open-access/author-compliance-tool.html.

3rd Confidential Review 25-Jan-2023
